# Supplementary material for: Bayesian inference across multiple models suggests a strong increase in lethality of COVID-19 in late 2020 in the UK
Source: PLoS One. 2021 Nov 24;16(11):e0258968. doi: 10.1371/journal.pone.0258968 (PMC8612566; doi:10.1371/journal.pone.0258968)
Supplement: S1 Appendix — Part A: Constitutive equations for the basic model. Part B: Intervention functions for Germany and France. Part C: Detailed plots of the MAP trajectories. (PDF) [file pone.0258968.s001.pdf]

S1 Appendix: Bayesian inference across multiple models suggests a strong increase in lethality of COVID-19 in late 2020 in the UK

A. Constitutive equations for the basic model

The differential equations determining the deterministic evolution of the mean occupation numbers of each compartment read

$$\dot{S}_i = -\lambda_i(t) S_i \tag{1a}$$
$$\dot{E}_i = -\gamma_E E_i + \lambda_i(t) S_i \tag{1b}$$
$$\dot{A}_i = -\gamma_A A_i + \gamma_E E_i - \tau(t)\pi_a A_i/\mathcal{N}(t) \tag{1c}$$
$$\dot{A}_i^Q = -\gamma_A A_i^Q + \tau(t)\pi_a A_i \tag{1d}$$
$$\dot{I}_i^a = \gamma_A \alpha_i A_i - \gamma_a I_i^a - \tau(t)\pi_a I_i^a/\mathcal{N}(t) \tag{1e}$$
$$\dot{I}_i^{a,Q} = \gamma_A \alpha_i A_i^Q - \gamma_a I_i^{a,Q} + \tau(t)\pi_a I_i^a/\mathcal{N}(t) \tag{1f}$$
$$\dot{I}_i^{s1} = \gamma_A (1-\alpha_i) A_i - \gamma_s I_i^{s1} - \tau(t)\pi_{s1} I_i^{s1}/\mathcal{N}(t) \tag{1g}$$
$$\dot{I}_i^{s1,Q} = \gamma_A (1-\alpha_i) A_i^Q - \gamma_s I_i^{s1,Q} + \tau(t)\pi_{s1} I_i^{s1}/\mathcal{N}(t) \tag{1h}$$
$$\dot{I}_i^{s2} = \gamma_s \sqrt{\mathrm{sifr}_i(t)} I_i^{s1} - \gamma_s I_i^{s2} - \tau(t)\pi_{s2} I_i^{s2}/\mathcal{N}(t) \tag{1i}$$
$$\dot{I}_i^{s2,Q} = \gamma_s \sqrt{\mathrm{sifr}_i(t)} I_i^{s1,Q} - \gamma_s I_i^{s2,Q} + \tau(t)\pi_{s2} I_i^{s2}/\mathcal{N}(t) \tag{1j}$$
$$\dot{I}_i^{m} = \gamma_s \sqrt{\mathrm{sifr}_i(t)} I_i^{s2} - \tau(t)\pi_m I_i^{m}/\mathcal{N}(t) \tag{1k}$$
$$\dot{I}_i^{m,Q} = \gamma_s \sqrt{\mathrm{sifr}_i(t)} I_i^{s2,Q} + \tau(t)\pi_m I_i^{m}/\mathcal{N}(t) \tag{1l}$$
$$\dot{R}_i = \gamma_a I_i^a + \gamma_s (1 - \sqrt{\mathrm{sifr}_i(t)})(I_i^{s1} + I_i^{s2}) - \tau(t)\pi_a R_i/\mathcal{N}(t) \tag{1m}$$
$$\dot{R}_i^Q = \gamma_a I_i^{a,Q} + \gamma_s (1 - \sqrt{\mathrm{sifr}_i(t)})(I_i^{s1,Q} + I_i^{s2,Q}) + \tau(t)\pi_a R_i/\mathcal{N}(t) \tag{1n}$$

with

$$\lambda_i(t) = \sum_j \beta_i C_{ij}(t)(A_j + I_j^a + I_j^{s1} + c I_j^{s2})/N_j \tag{2}$$

and

$$\mathcal{N}(t) = \sum_i \left[\pi_a(S_i + E_i + A_i + I_i^a + R_i) + \pi_{s1} I_i^{s1} + \pi_{s2} I_i^{s2} + \pi_m I_i^m\right]. \tag{3}$$

The stochastic differential equations underlying our computation of the likelihood follow in a linear noise approximation of the corresponding master equation. Denoting the stochastic compartment numbers also as $S_i, A_i, \ldots$ (for notational simplicity), a stochastic noise term is

added to each term in Eq. (1). This leads to

$$\dot{S}_i = -\lambda_i(t)S_i - \sqrt{\eta_{\text{infect}}\lambda_i(t)S_i}\zeta_i^{(0)}(t) \tag{4a}$$

$$\dot{E}_i = -\gamma_E E_i + \lambda_i(t)S_i - \sqrt{\gamma_E E_i}\zeta_i^{(1)}(t) + \sqrt{\eta_{\text{infect}}\lambda_i(t)S_i}\zeta_i^{(0)}(t) \tag{4b}$$

$$\dot{A}_i = -\gamma_A A_i + \gamma_E E_i - \tau(t)\pi_a A_i/\mathcal{N}(t) - \sqrt{\gamma_A A_i}\zeta_i^{(2)}(t) + \sqrt{\gamma_E E_i}\zeta_i^{(1)}(t) - \sqrt{\eta_{\text{test}}\tau(t)\pi_a A_i/\mathcal{N}(t)}\zeta_i^{(3)}(t) \tag{4c}$$

$$\dot{A}_i^Q = -\gamma_A A_i^Q + \tau(t)\pi_a A_i - \sqrt{\gamma_A A_i^Q}\zeta_i^{(4)}(t) + \sqrt{\eta_{\text{test}}\tau(t)\pi_a A_i/\mathcal{N}(t)}\zeta_i^{(3)}(t) \tag{4d}$$

etc.

with white noise processes satisfying $\langle\zeta_i^{(\mu)}(t)\rangle = 0$ and $\langle\zeta_i^{(\mu)}(t)\zeta_j^{(\nu)}(t')\rangle = \delta_{ij}\delta_{\mu\nu}\delta(t-t')$. In order to account for sources of noise otherwise not resolved in the model, we include overdispersion factors in the noise terms relating to infections (η_{infect}), to testing (η_{test}), and to deaths (η_{death}). The latter is included in transitions from $I^{s2,Q}$ to $I^{m,Q}$ and from I^m to $I^{m,Q}$.

B. Intervention functions for Germany and France

The timing and type of the modeled interventions for Germany and France are listed in Tabs. 1 and 2 (analogous to Tab. 1 of the main text for the UK).

dates	type	control parameters
before 2020-03-09	before lockdown (reference)	$a(t) = 1$, $s(t) = 0$
2020-03-09 to 2020-03-23	imposition of lockdown	linear decrease of $a(t)$, new value $s(t)$
2020-03-23 to 2020-06-22	easing of / increasing non-compliance with lockdown	linear increase of $a(t)$, linear change of $s(t)$
2020-06-22 to 2020-11-02	lockdown lifted	new values of $a(t)$ and $s(t)$
inferred	increase of contacts / infectiousness in autumn	tanh-shaped increase of $a(t)$ and change of $s(t)$, centre and width to be inferred
2020-11-02 to 2020-12-14	lockdown "light", local interventions	new values for $a(t)$ and $s(t)$
after 2021-12-14	national lockdown	new values for $a(t)$ and $s(t)$

Table 1: Interventions considered in model GER-C. Dates are always rounded to the closest Monday.

dates	type	control parameters
before 2020-03-08	before lockdown (reference)	$a(t) = 1$, $sh(t) = 1$, $s(t) = 1$
2020-03-18 to 2020-05-13	first lockdown	new values for $a(t)$, $sh(t)$, $s(t)$
2020-05-13 to 2020-06-14	easing of lockdown	linear change for $a(t)$, $sh(t)$, $s(t)$
2020-07-03	start of school holiday	change in $s(t)$
2020-06-14 to 2020-07-11	restrictions are lifted	linear change $a(t)$, $s(t)$
2020-07-11 to 2020-11-01	more easing of restrictions, schools are reopened	new values for $a(t)$, $s(t)$
inferred	autumn	tanh-shaped increase for $a(t)$
2020-11-27	effect before lockdown is imposed	change in $a(t)$
2020-11-01 to 2020-11-27	second lockdown, primary schools never close	change in $a(t)$, $sh(t)$
2020-11-27 to 2020-12-15	easing of second lockdown	new values for $a(t)$, $sh(t)$
after 2020-12-15	introduction of nightly curfew	new values for $a(t)$, $sh(t)$

Table 2: Interventions that are considered in model FRA-C. The $a(t)$ refers to scaling of contacts for non-school contacts, $s(t)$ refers to the scaling of school contacts, and $sh(t)$ refers to the scaling of the shielding vector.

C. Detailed plots of the MAP trajectories

In this appendix, we provide detailed plots for each of the model variants, showing the trajectories corresponding to the MAP parameters along with data for cases, fatalities and testing.

C.1. UK

The results for all model variants for the UK are shown in Figs 1-12.

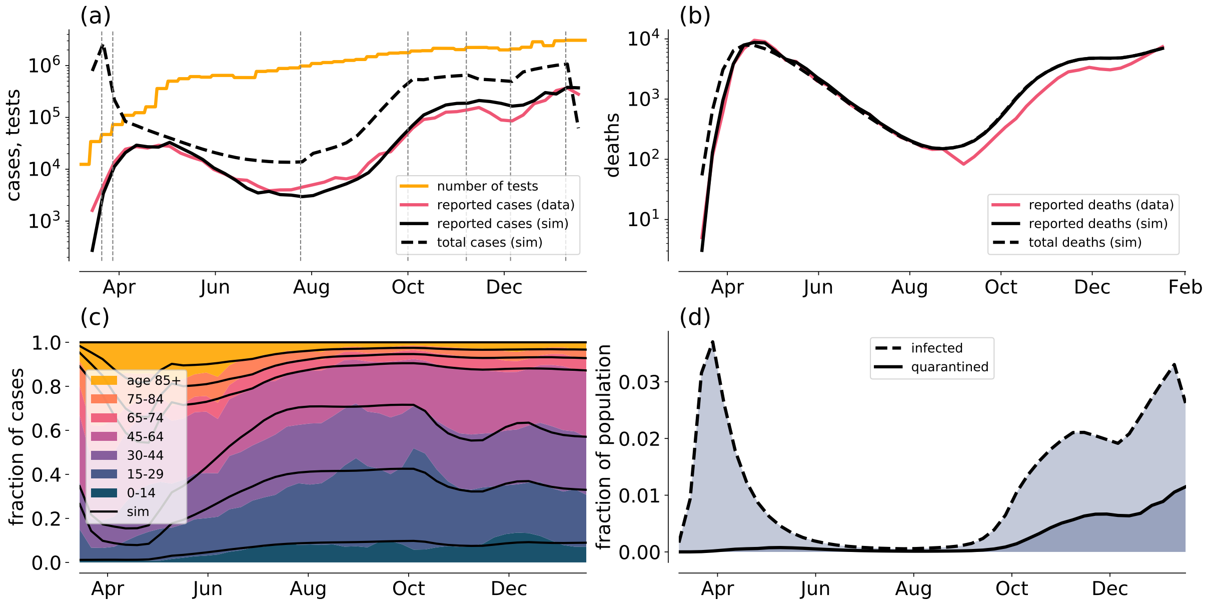

Figure 1: Plots of the MAP trajectories for model UK-A0. Expected trajectories are shown in black and labelled as 'sim' (solid for observable quantities, dashed for hidden quantities). Data are shown in colour. Panel (a) shows weekly diagnosed case numbers along with the total number of new infections and the number of tests performed. The vertical lines indicate times where interventions change. Panel (b) shows weekly deaths. Panel (c) shows the distribution of ages among the weekly new cases. Data are shown in colour, stacked and scaled to add up to 1. Analogously, boundaries between age groups in the simulation are shown in black. Panel (d) shows the prevalence as a fraction of infected (dashed) and quarantined (solid) individuals in the total population.

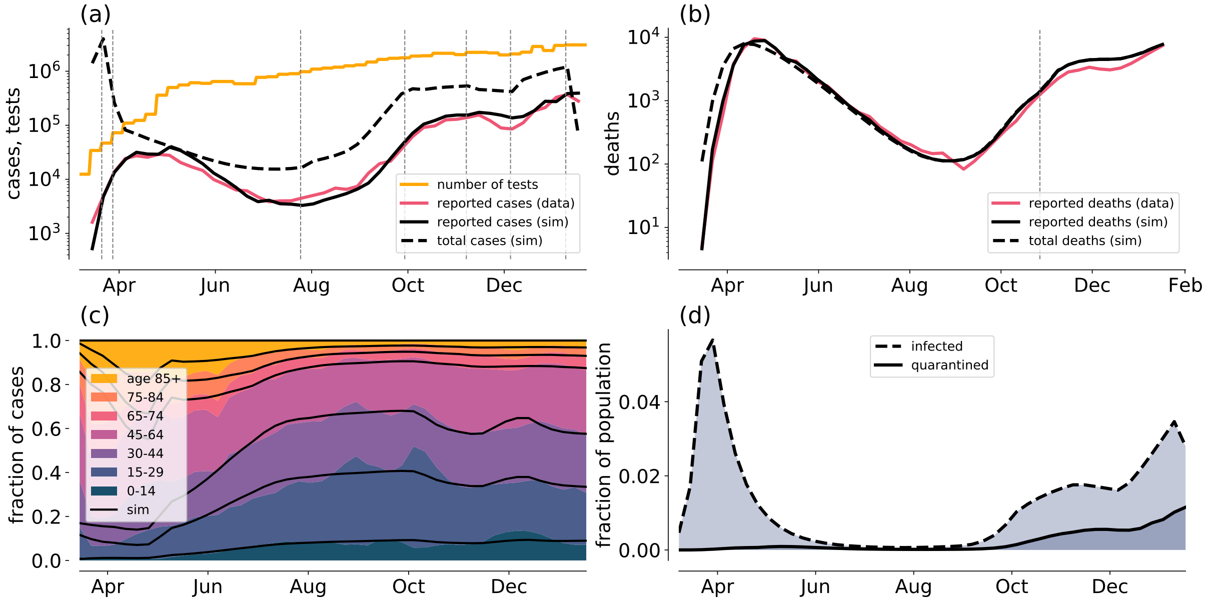

Figure 2: MAP trajectories for model UK-A1, as above. Additionally, the inferred time of change in IFR is indicated as a vertical line in panel (b).

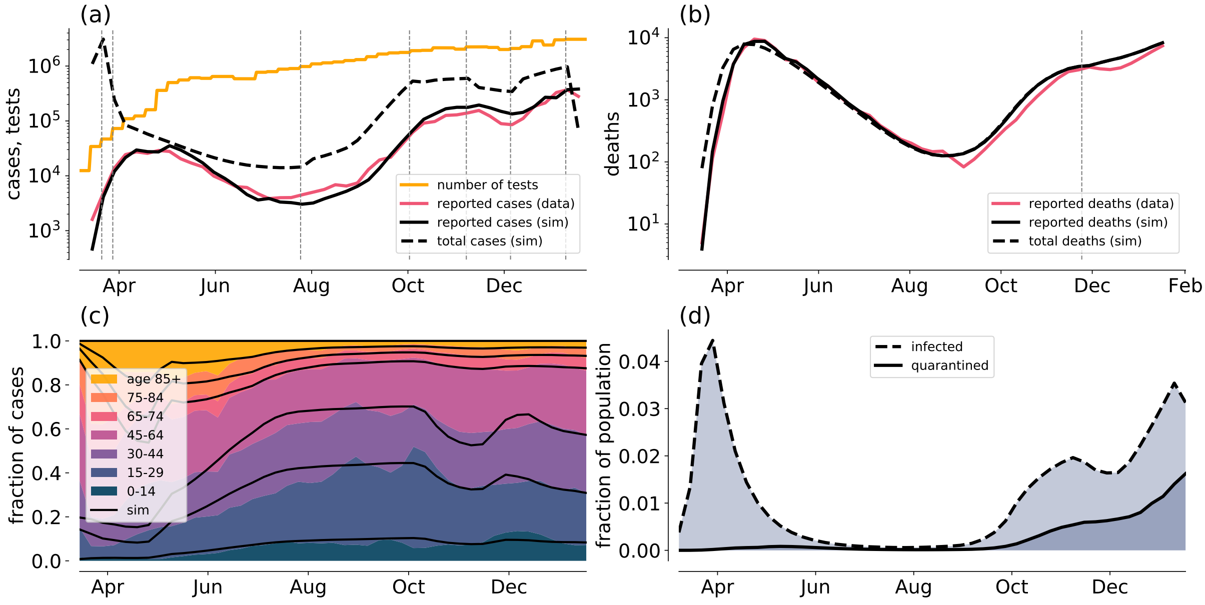

Figure 3: Model UK-A2

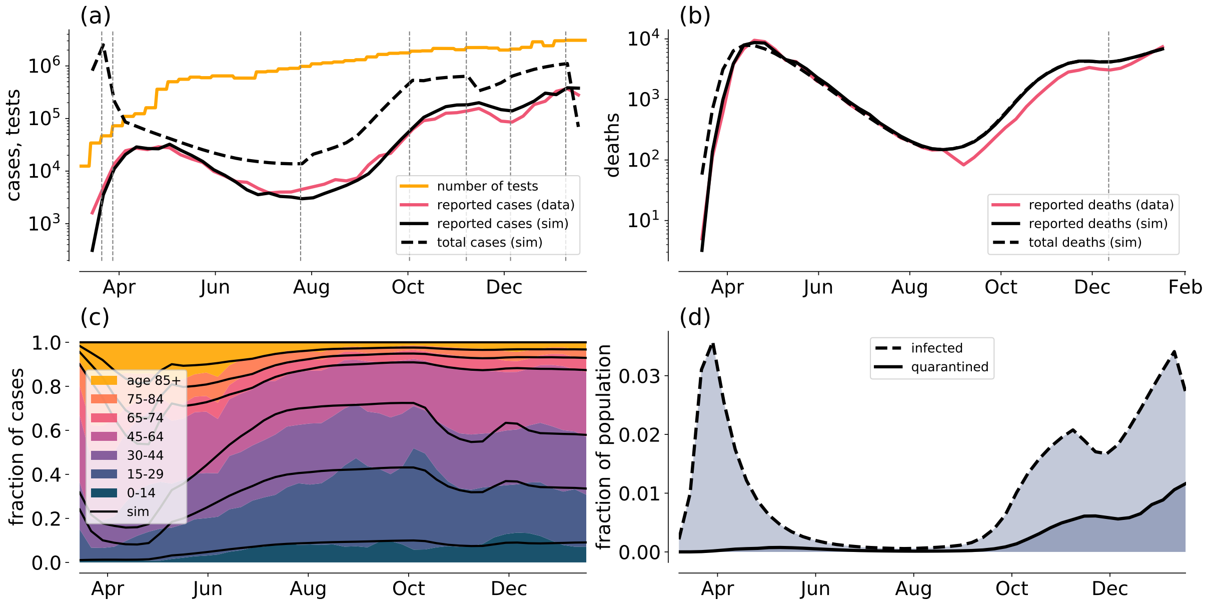

Figure 4: Model UK-B0

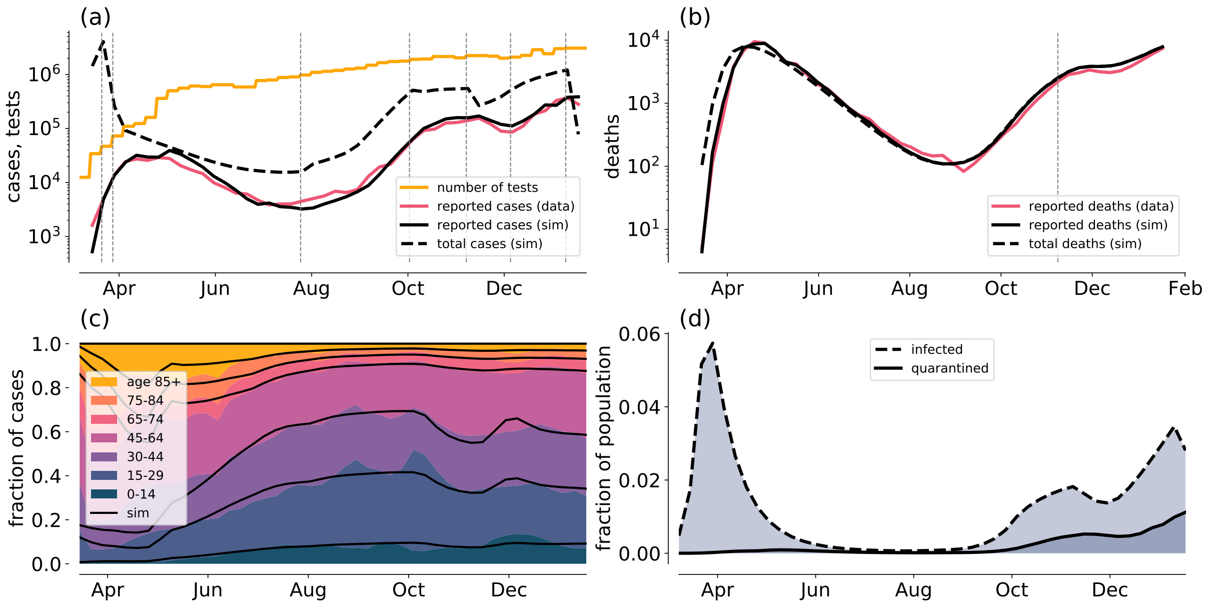

Figure 5: Model UK-B1

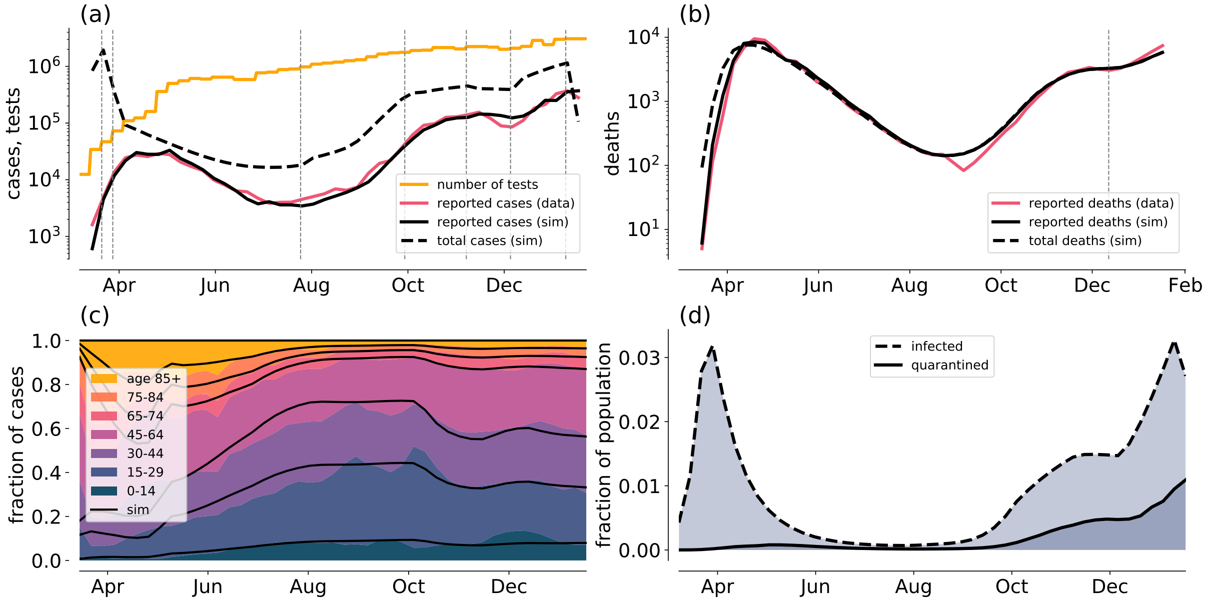

Figure 6: Model UK-C0

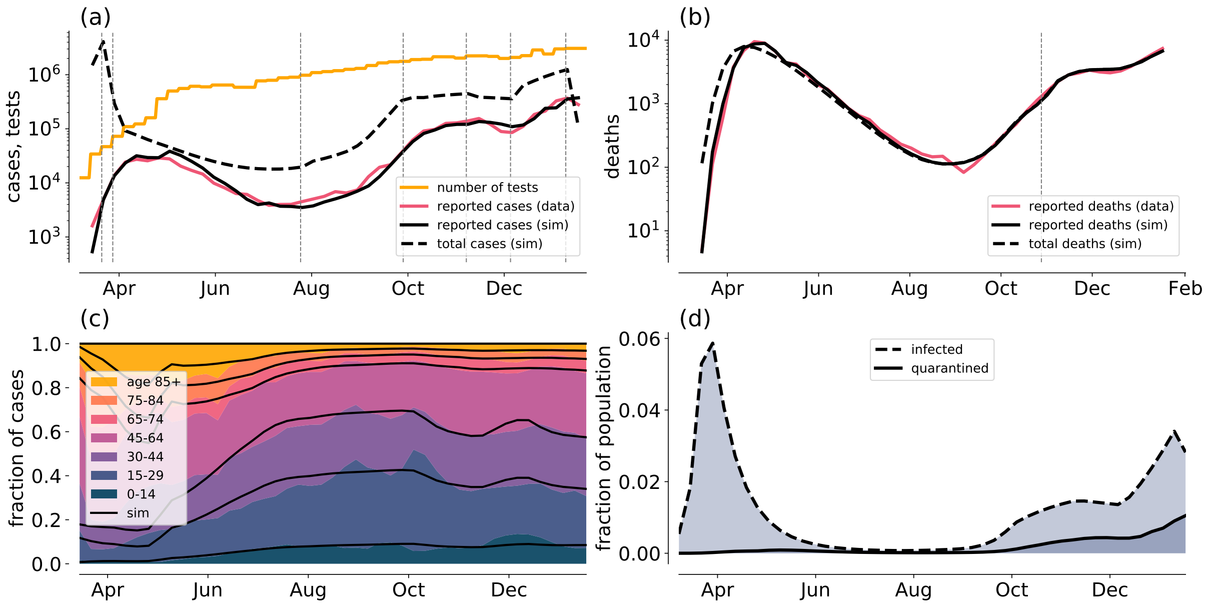

Figure 7: Model UK-C1

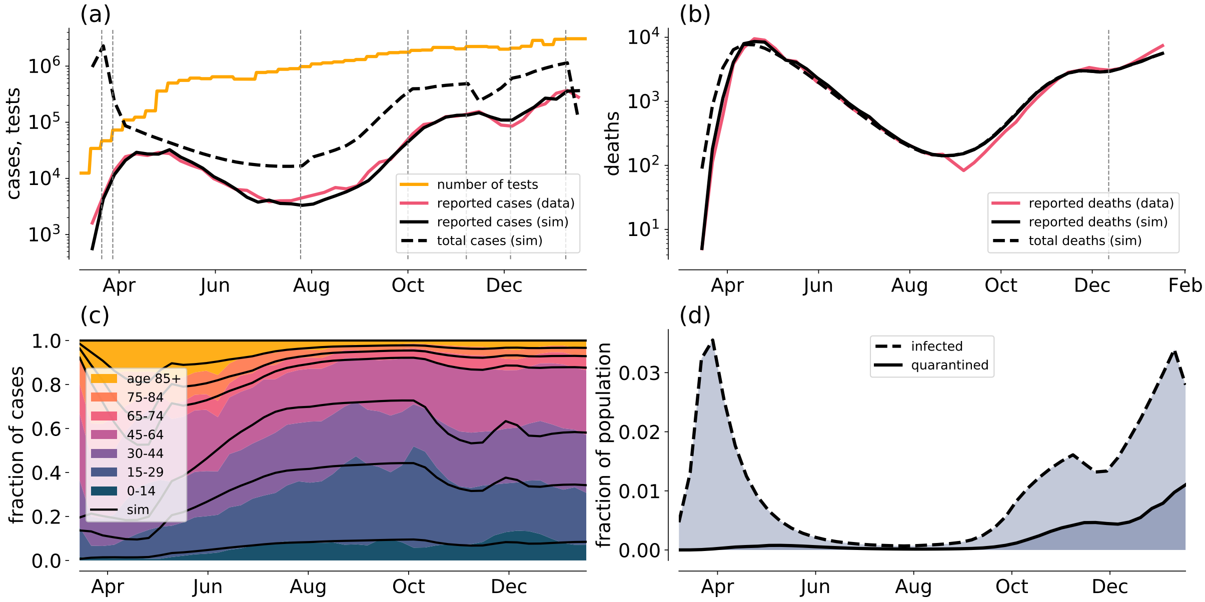

Figure 8: Model UK-BC0

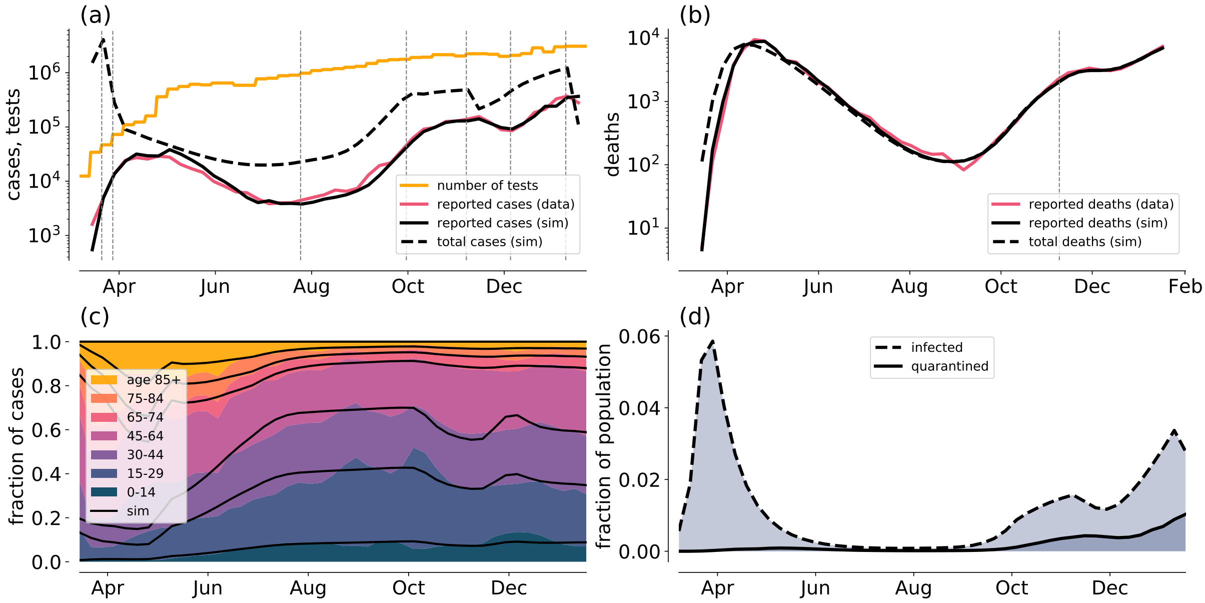

Figure 9: Model UK-BC1

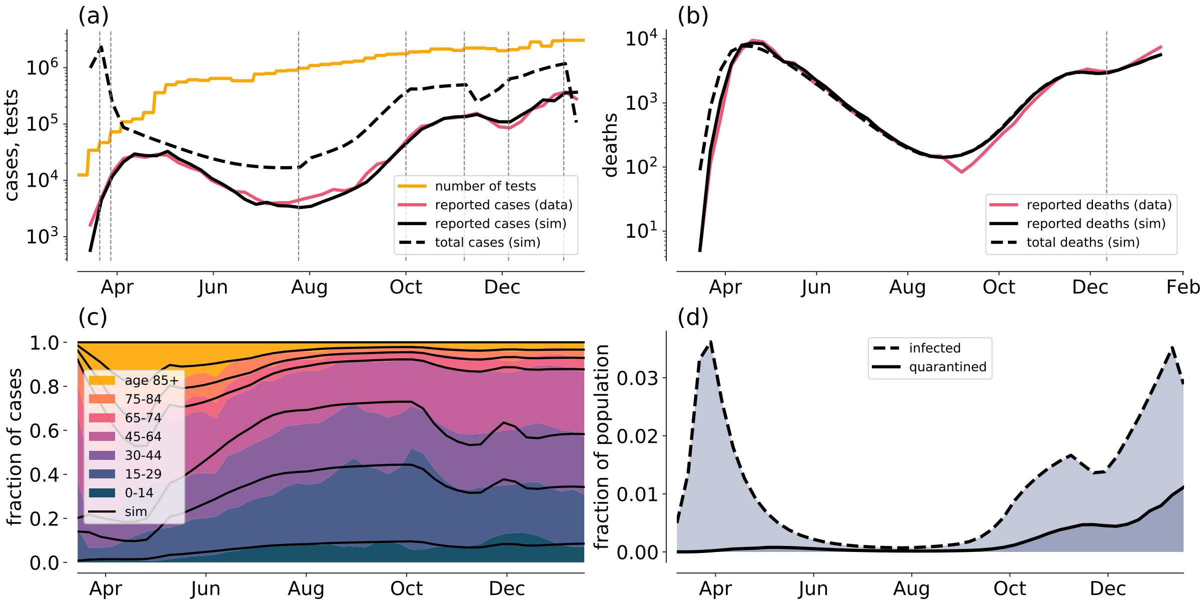

Figure 10: Model UK-TT0

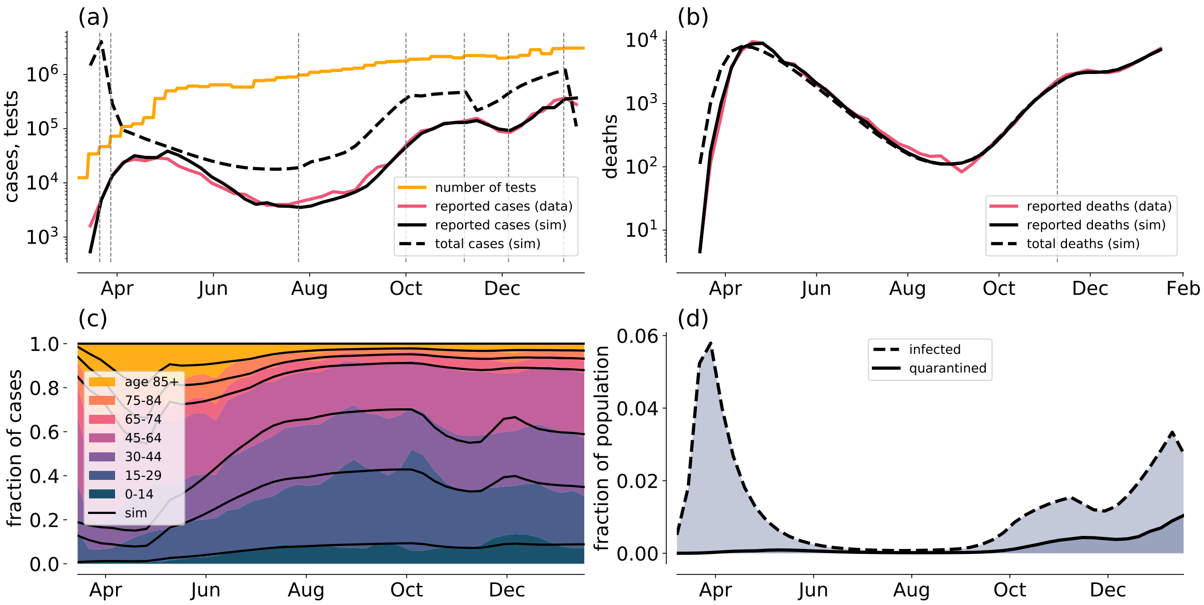

Figure 11: Model UK-TT1

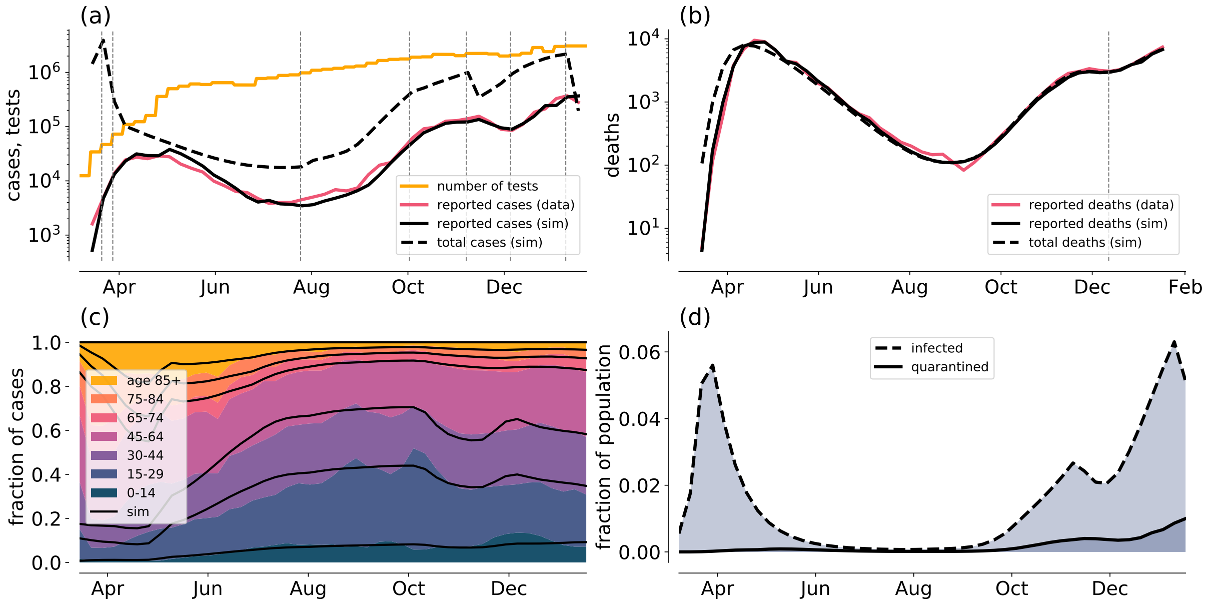

Figure 12: Model UK-P0

C.2. Germany

The plots for model variants C0 and C1 for Germany are shown in Figs. 13 and 14.

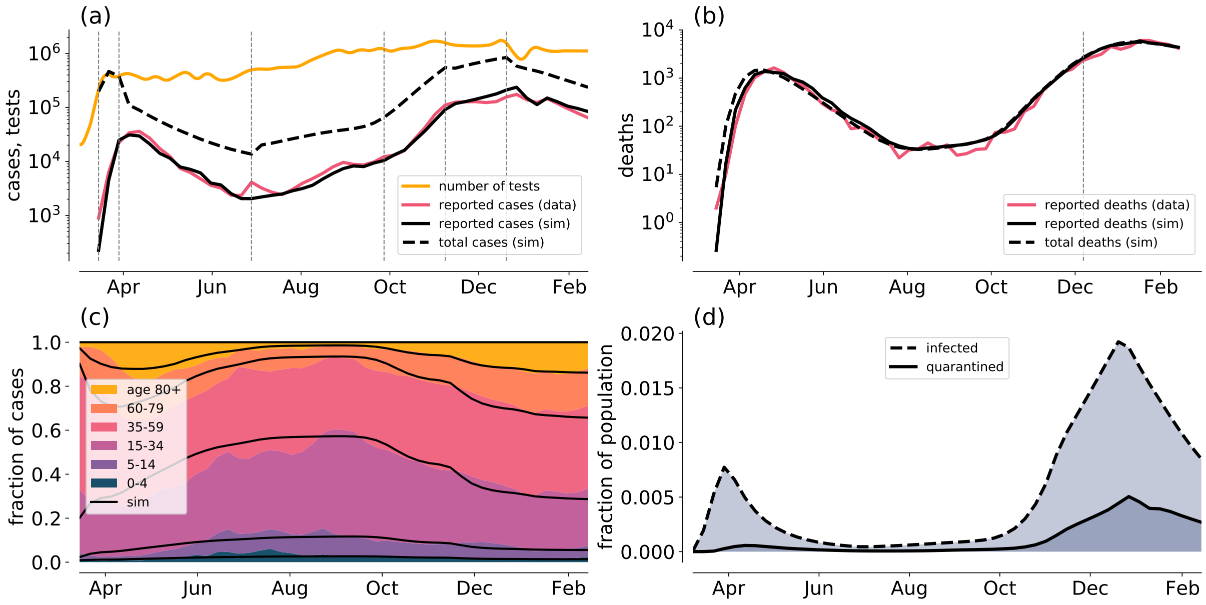

Figure 13: Model GER-C0

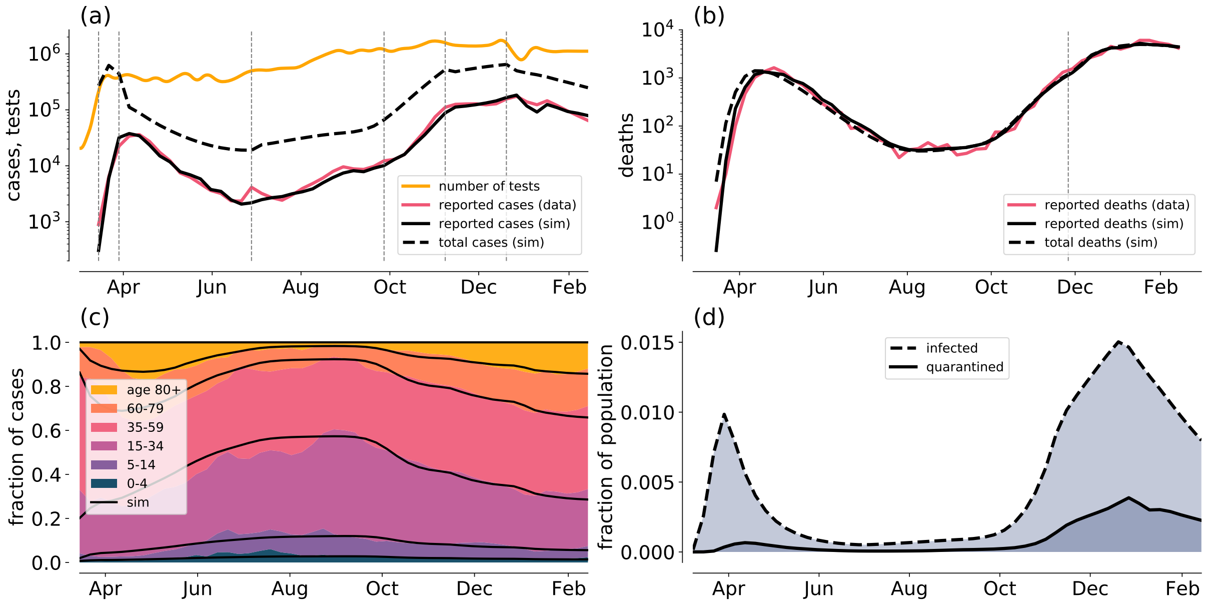

Figure 14: Model GER-C1

C.3. France

The plots for model variants C0 and C1 for France are shown in Figs. 15 and 16.

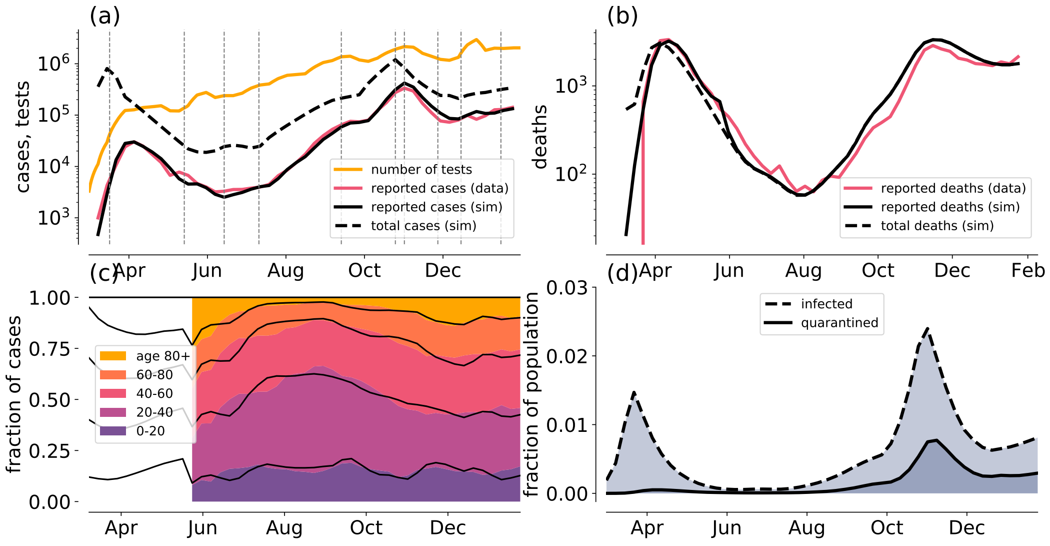

Figure 15: Model FRA C0.

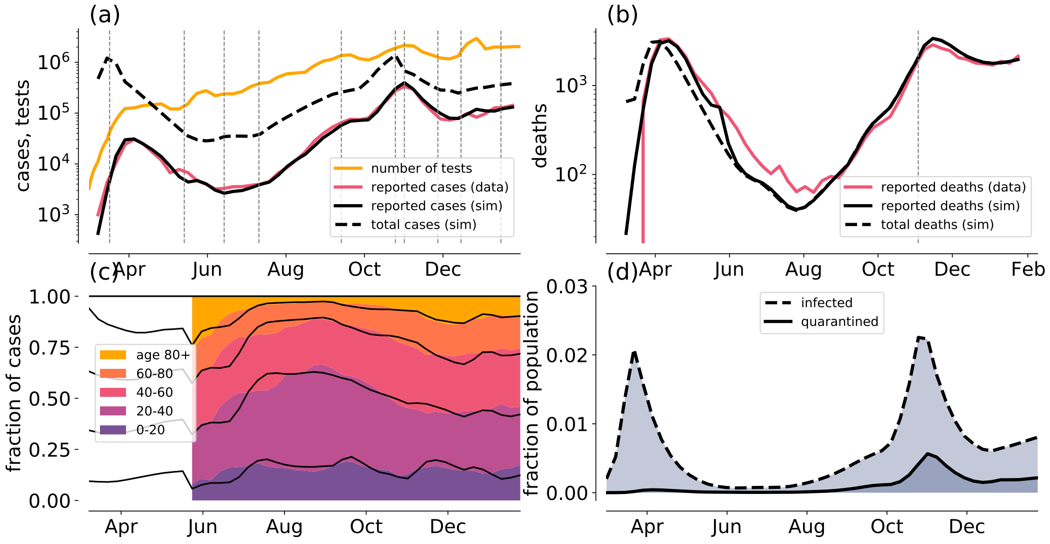

Figure 16: Model FRA C1.
