# Supplementary material for: Bayesian inference across multiple models suggests a strong increase in lethality of COVID-19 in late 2020 in the UK
Source: PLoS One. 2021 Nov 24;16(11):e0258968. doi: 10.1371/journal.pone.0258968 (PMC8612566; doi:10.1371/journal.pone.0258968)
Supplement: Attachment — Submitted filename: reply.pdf [file pone.0258968.s003.pdf]

Manuscript PONE-D-21-08131

Dear Professor Shaman,

Thank you for your message from 26th April, including your own thoughtful comments on our manuscript and the refereesąŕ assessment. Please find below a detailed reply to all the points raised. In our updated manuscript we have clarified these points. Moreover, we have improved our method, such that we can now provide a complete list of confidence intervals for all inferred parameters as well as a direct quantification of the model evidences, as suggested by reviewer 2.

We have also fixed a few typos and minor inaccuracies (such as the fact that B.1.1.7 is by now no longer dominant). All changes are highlighted in the attached diff-file (page numbers quoted here refer to the new plain manuscript file).

Yours sincerely,

Patrick Pietzonka, Erik Brorson, William Bankes, Michael E. Cates, Robert L. Jack and Ronojoy Adhikari

(Note: Comments from the Editor and referees are printed in *blue*, our response in black)

Reply to the Editor

Editor: I'd also like the authors to explore whether fixing most of the π parameters, e.g. π_{Is}, affects the results. This seems to assume that testing rates among the symptomatic didn't change over time, or merely that those changes will all be handled by the π_a term (which really appears intended to represent tracing and capture of asymptomatics). Is this assumption a problem for constraining fluctuating testing capacity and demand for testing rates over time? Evidence suggests that ascertainment rates increased over time, which would work against the rising IFR reported here.

We agree that determination of the time-dependent ascertainment rate is vital, in order to arrive at our conclusions. In our model of the testing process, the total number of tests is fixed by the data, and the priorities (such as π_{Is} and π_a) describe which individuals came forward to be tested. The priorities are relative quantities so the choice $\pi_{Is} = 1$ is a normalisation condition, it does not restrict the model behaviour.

Within this framework, the ascertainment rate depends on two things: how effectively the tests are targeted towards symptomatically infected individuals, and whether there is enough capacity to cover all of them. (These are both important effects in reality, which are captured in the model.) The result is an increasing ascertainment rate, if the testing capacity rises faster than the number of cases. The ascertainment rate in our model (evaluated in retrospect as the fraction of individuals who get diagnosed before they recover) is generally increasing to up to 45%, but is reduced again around the two peaks of the second wave.

We agree that a significant assumption of the model is that the testing priorities are independent of time (except in the model variants TT0, TT1, and P0), but the model can still capture the changing ascertainment rate. We have slightly revised the Sections "Introduction", "Testing"

and "Discussion" to clarify how we account for a changing ascertainment rate.

. . . What do the raw case fatality rates show (when convolving cases forward to deaths)? Do they rise as well? How about the hospitalization fatality rates? Do they rise or fall in the study period? This can be pulled from observations and provide an indication as to whether clinical treatment has improved.

Unlike the IFR, the case fatality rate (CFR) is not a parameter of our model and thus cannot be inferred in a Bayesian fashion. An empirical analysis of changes in CFR has been performed by Wallace and Ackland in Ref. [6], ("convolving cases forward to deaths", as you suggest). They have shown that indeed the CFR did rise as well, which prompted us to analyse this finding in the light of changing testing behaviour. Also see their more recent paper, added as Ref. [7].

We have opted not to make use of hospitalisation data in our study, because the hospitalisation process itself is subject to uncertainties stemming from the time-dependent pressure on the health care system. Our focus was instead on overall changes of the fatality rate. We could not find any data on changes in the hospitalization fatality rate in the UK for the period of interest in our study. There is a cohort study dating until September [Gray et al., `https://doi.org/10.1016/j.lanepe.2021.100104`], which shows a decrease around May (changes around this time have not been the focus of our study). This could be an indication of seasonal effect, or it could be due to less severe cases being admitted to hospital after the first wave subsided.

. . . Is your main finding the result of a poorly constrained total number of infection (i.e. the ascertainment rate)?

The main finding is that the inferred IFR is larger in the later part of the studied time period (Dec/Jan).

In practice, the difficulty is how to account for the large number of deaths in Dec/Jan. An increased IFR is a natural way to do this (as in model BC1, for example). However, there would be other possibilities – for example, a huge number of undiagnosed cases in December could account for the increased deaths, which corresponds to a lower ascertainment rate. The model P0 tests this hypothesis: Fig 5 shows that the inferred behaviour in this case is not consistent with other available data.

Hence, while we agree that the number of infections is hard to characterise, we believe that our finding is still robust.

Reply to the First Referee

The paper was a pleasure to read. The summary is excellent. The clear way in which you present your models and your results gives me total confidence in your work. I recommend that it should be published. It is valuable not only for its results but also because it provides a nice demonstration of your methods and of your PyRoss software package and will help others to build on this. I only have two suggestions 1) please make the code available online and 2) it would be nice if you could give the reader access to the MAP values for all inferred parameters. I would have liked to check whether they look believable, but I also think I would have learned

something from them.

We thank the reviewer for this positive evaluation.

As requested, we now have made a Jupyter notebook for the model variant BC1 available online, see `https://github.com/ppietzonka/pyross-ifr-change`. This model variant is the most detailed one, other variants follow readily. The notebook employs our software package PyRoss, which is available on `https://github.com/rajeshrinet/pyross`.

We have added a spreadsheet with all details on model parameters (including the MAP parameters) as supplementary information.

Reply to the Second Referee

Dear editor,
the manuscript analyses public COVID-19 data in the UK, France and Germany using a SEIR type compartmental model. The text is clearly written and the proposed models interesting and well thought-out. The authors conclude that the IFR in the UK and Germany was higher in the end of 2020 by a factor of around 2 when compared to the first semester. They also conclude that this increase precedes the widespread circulation of the new major SARS-CoV-2 variant B.1.1.7. I consider this a very strong claim which is not convincingly backed by the methods and analyzed data presented in the current manuscript. I cannot recommend publication unless the authors are able to provide further details and/or revise their analysis in order to better substantiate their main conclusion.

We wish to thank the reviewer for the careful reading of our manuscript and the suggestions. We hope that with the present revision, in particular the new Sec. 5, we now meet all the concerns of the reviewer. Please see below for a point-by-point reply.

MAJOR POINTS

1 - The authors make use of the PyRoss package in order to evaluate their posterior and quote maximum posterior values for their parameters. Although I understand this package was already discussed in more detail in a previous manuscript, the authors should explain here in more detail the methods involved in their analysis. For instance, are the quoted values for the IFR change marginalized over all other model parameters? Was the posterior sampled with MCMC methods or is it the maxima found with a simpler scheme? If the latter, what is the justification and how accurate is it?

The central estimates for the IFR change are the ones that maximise the posterior probability, as computed by the CMA optimizer, which is an evolutionary algorithm for global optimization. To characterise the uncertainty, we used for each of the model variants a Gaussian approximation to the posterior, obtained by computation of the Hessian of the posterior. The resulting uncertainties are marginalised over other parameters, within this approximation. Using MCMC was not an option here, although it was used for the simpler model considered in [9]. The reason is that a single likelihood computation is quite expensive (around 30 seconds on a single core). The parameter space is high-dimensional (around 70 inferred parameters, see Table 2): combined with the expense of a likelihood computation, this makes MCMC intractable.

The Gaussian approximation of the posterior is consistent with its observed behaviour (see the new Fig 6), and the MCMC results of previous work [9] were also found to be reasonably consistent with a Gaussian distribution.

(We note in passing that a simpler approximation to the model likelihood might make MCMC more tractable, at the expense of a different set of (uncontrolled) errors. In particular, the likelihood used here accounts for correlations between observed data in different weeks, which have significant consequences in this analysis.)

2 - Why do the authors refrain from quoting confidence intervals CI (ideally highest density intervals) in most of their results? The claim that "the calculation is tedious" seems unjustifiable, as the discussed method based on the Hessian seems to be the very well established method of using the Fisher information Matrix. In particular the Li et al. 2020 paper which describes the package PyRoss discusses the implementation of both MCMC and Fisher information Matrix methods. In order to help make sense of the main results for the IFR I consider estimations of the marginalized CI for all main results imperative. Ideally one should show plots of the posterior for the IFR (before, after and mean IFR for the models without a change) for at least some models.

We have answered this point by computing the Hessian of the log-posterior for all model variants, see the new Sec. 5. This provides a Gaussian approximation to the posterior, and it enables estimation of the (Bayesian) model evidence, which is now shown in Table 2.

The Hessian is estimated by finite differences. Given the large number of inferred parameters, this computation requires a lot of (expensive) likelihood computations, which is somewhat tedious. (Also, some care is required to ensure reliable finite-difference estimates, including step sizes and some tolerances that are used in the likelihood computation).

The resulting marginalised confidence intervals are now listed in Tab. 2 for the parameters concerning the IFR change, and in full detail for all inferred parameters in the spreadsheet attached as supplementary information.

Using the Gaussian approximation, we can now analyse the posterior distribution of the parameters relating to the IFR, as suggested by the referee. In the new Fig. 7, we show samples of the time-dependent IFR with parameters drawn from the posterior distribution.

. . . Also, in the 2 cases for which uncertainties are shown, they seem quite narrow, specially for the German IFR factor. Is that expected after marginalization over dozens of parameters?

The inferred confidence intervals are indeed quite narrow, but not unusually so. We do marginalise over dozens of parameters, but still the number of data points is much larger (>600), such that deviations away from the optimal set of parameters can be assigned a small likelihood. Note that we quote a single standard deviation, the 95% confidence intervals would be twice as large. This still allows for considerable variation in the potential courses of the change of IFR, as illustrated in the new Fig. 7.

Tab. 2 suggests that small confidence intervals for the parameters of the IFR change appear to be linked to model variants with a step-like change in the IFR (models A1 and C1), which we had also used for Germany (and France). These models seem to be fitting short-lived fluctuations in cases and mortalities, which leads to a more sharply peaked local Gaussian approximation.

However, a step-like change in the IFR is a simplistic assumption, and should be considered *a priori* less likely than the more realistic smooth change of the other model variants.

3 - The authors choose not to make a full model-comparison using the Bayesian evidence and instead rely on a simple analysis of the maxima of the Posterior. I think that if the full evidence cannot be computed due to computational complexity, at least some better quantitative proxy for it should be employed. Alternatives are the Akaike or Bayesian Information Criteria, which are fast to compute. Or at the very least a Goodness of Fit test.

As noted in the response to point 2 above, we have computed Gaussian approximations to the posterior, from which we now estimate the model evidence. The conclusion (see the revised Table 2) is that the differences in log-evidence between model variants are close to the difference in the log-posterior, and are large enough to prefer the models with an IFR-change. Simple estimates based on BIC/AIC lead to similar conclusions, but estimation of a Gaussian posterior should be a more accurate way of incorporating posterior uncertainty into the evidence.

4 - The main conclusion is that the IFR seems to have increased by a factor of around 2 in the end of 2020. Nowhere in the text however are the actual inferred values for the IFR (before and after) written down. Nor is there a discussion on how these estimates compare with other IFR estimates in the literature (for the UK or other countries), specially with those which do not rely on similar modelling.

We now list all inferred parameters in the spreadsheet in the appendix, including the IFR before the change (the IFR after the change follows by simple multiplication). Note, however, that the IFR is age-dependent and can therefore not be summarised in a single number. We have sought to limit the number of additional fit parameters by having just a single factor of change that applies to all age cohorts (except for the youngest ones where fatalities are extremely rare). The new Fig. 7 shows the IFR before and after the change for the relevant age groups.

Published estimates for the IFR vary widely by more than a decade (see e.g. the new Ref. [27] for a worldwide metastudy, or [28] specifically for Germany). Our inferred values for the IFR are within the range of variation reported there. This might, inter alia, be due to differences in what counts as an infection: A clearly symptomatic case or a viral load at the threshold of being detectable in an infection. Depending on this definition, a single set of data could be fit by either a high number of asymptomatic cases that are little infectious or vice versa (as long as the immunity of "recovered" asymptomatic cases is not yet significant). This corresponds to a ridge in the likelihood (also known as a "soft mode" in the parameter space), and is also present in our model. Therefore, while the absolute values of the IFR cannot be inferred precisely, its change shows up clearly.

We now discuss these matters briefly in the new Sec. 5 and in the updated Sec. "Discussion".

5 - As the authors claim, for IFR estimates one needs accurate estimates of the total number of cases, including undiagnosed ones. As the authors point out a bias in the estimated number of cases in different months can affect their inferred IFR increase. This point deserves a more careful discussion as any IFR estimate hinges strongly on the estimation of the total number of cases. I would like the authors to discuss in more detail how the ONS random asymptomatic testing was conducted and how reliable are their estimates. How does it compare with other random seroprevalence surveys (in the UK or elsewhere)?

The data from the ONS infection survey is based on (anonymised) PCR tests for a suitably random sample of the UK population.

We consider the data from the infection survey of Office for National Statistics (an institution of the UK government) to be reliable, methods and further information can be found here: https://www.ons.gov.uk/peoplepopulationandcommunity/healthandsocialcare/conditionsanddiseases/methodologies/covid19infectionsurveypilotmethodsandfurtherinformation#14-day-estimates. We refrain from a discussion of their methods, since this would lead beyond the scope of the present paper.

Comparable surveys are usually seroprevalence studies using antibody tests. These may not be directly comparable to the data from the infection survey, since antibody levels may vary depending on age and time since infection.

The purpose in our study was not to provide absolute numbers for the dark figure of cases and thus the IFR, this would require extensive use of randomised testing data. We rather want to show how relative changes in the IFR can be inferred using only data for cases and mortalities, which are most readily and recently available for most countries and regions. The quoted data from the ONS infection survey serve merely as a consistency check for our models, *a posteriori*.

MINOR POINTS

1 - There have been estimates in the literature of the time lag between contagion and mortality and between development of symptoms and mortality. How do these estimates compare with the values in the models used?

The parameters and estimates we have used for the latent and incubation period are in line with common values in the literature (see https://arxiv.org/abs/2006.01283 for an overview), and the values used in similar modeling studies (e.g. by Birrell et al., Ref. [20]). See the added note in the paragraph "Infection dynamics" of Sec. 2.

For the time between onset of symptoms and deaths, we infer a mean of 33 days for the UK (with a standard deviation of just 0.5 days). This is considerably more than the 18 days quoted in Ref. [21]. As we now note on p. 14, this could be due to the specific way in which deaths are reported in Ref. [11]. This inferred value is fairly robust across model variants and unrelated to our choice of the prior. (The prior distribution is very loose, and approximately the same value is inferred if we set the prior mean to half the period. Fixing the period to just 14 days results in a strongly reduced model evidence.)

2 - Some variables are not clearly defined in the text, which may hinder understanding readers less familiar with SEIR models. For instance: ϕ_X, γ_A, γ_E, $a(t)$, $s(t)$.

We have added these definitions and a few further explanations in the paragraphs "infection dynamics" and "contact behaviour" of Sec. 2.

3 - For the models with allow a step-change in IFR values the window for change has a narrow 2-week sigma. Is it possible that by using a much broader window the models could have preferred a much different change time? In other words, since there has already been claims of a change of IFR in the period considered, could this "a posteriori information" be biasing the models?

This is a valid point. We had been motivated by previous reports, based on a non-Bayesian analysis. We were specifically interested in changes in the IFR around the time when the alpha variant became dominant, and we were deliberately setting this window such that we would not pick up other seasonal changes (possibly in spring). This could, in principle, lead to the kind of "confirmation bias" described by the referee. In practice, however, the 2-week sigma does not impose a strong bias towards a specific date. For instance, a 4-week deviation from the prior mean would correspond to a change in the log-prior of just two, this is very little compared to the changes in posterior and evidence that qualify the model variants with changing IFR. Moreover, for the models for France and Germany we had no such prior information, nonetheless we detect similar evidence for a changing IFR.

4 - The full list of parameters for the models (at least A0) should be more explicitly written down in an appendix.

The full list of parameters is now included in the spreadsheet attached as supplementary information.
